# Consumption of non-nutritive sweeteners by pre-schoolers of the food and environment Chilean cohort (FECHIC) before the implementation of the Chilean food labelling and advertising law

**DOI:** 10.1186/s12937-020-00583-3

**Published:** 2020-07-10

**Authors:** Carolina Venegas Hargous, Marcela Reyes, Lindsey Smith Taillie, Carmen Gloria González, Camila Corvalán

**Affiliations:** 1 Institute of Nutrition and Food Technology, University of Chile, Av. El Líbano 5524, Macul, Casilla 138-11, Santiago, Chile; 2 Carolina Population Center, University of North Carolina at Chapel Hill, 123 West Franklin St., Suite 210, Chapel Hill, 27516 NC United States

**Keywords:** Preschool children, Non-nutritive sweeteners, Sweetening agents, Chilean law of food labelling and advertising, Food reformulation

## Abstract

**Background:**

Consumption of non-nutritive sweeteners (NNS) is becoming increasingly more frequent, particularly in the context of obesity prevention policies. The aim of this study was to describe the consumption of NNS in an ongoing cohort of pre-schoolers (4–6-year-old) before the implementation of the Chilean Food Labelling and Advertising Law, identify sociodemographic and anthropometric characteristics associated with their consumption, and describe the main dietary sources of each NNS sub-type.

**Methods:**

In 959 low-medium income pre-schoolers from the Food and Environment Chilean Cohort (FECHIC), dietary data from a single 24-h recall was linked to NNS content information obtained from packaged foods (*n* = 12,233). The prevalence of NNS consumption was estimated by food source and characterized by child and maternal sociodemographic and anthropometric variables. Intakes and main dietary sources were described for the six most prevalent NNS in Chile: Sodium Cyclamate, Saccharin, Aspartame, Acesulfame Potassium, Sucralose, and Steviol glycosides.

**Results:**

Sixty-eight percent of the pre-schoolers consumed at least one source of NNS on the day of the dietary recall; most of them consumed NNS from foods and beverages (*n* = 532), while only 12% (*n* = 119) also consumed table-top sweeteners. The prevalence of NNS consumption was significantly higher among children whose mothers had a high educational level compared to children whose mothers did not complete high school (*p* < 0.05); however, it did not differ by any other variable studied. The highest intakes of NNS were observed for Aspartame [2.5 (1.4–3.7) mg/kg per consumer], followed by Sodium Cyclamate [1.6 (1.3–2.6) mg/kg per consumer] and Steviol glycosides [1.2 (0.2–2.1) mg/kg per consumer]. Beverages were the only food group that contributed to the intake of the six NNS studied, accounting for 22% of the overall intake of Saccharine and up to 99% of Aspartame intake.

**Conclusions:**

Before the implementation of the Food Labelling and Advertising Law, NNS consumption was highly prevalent among a cohort of low-middle income Chilean pre-schoolers. Continuous monitoring of NNS consumption is essential given potential food reformulation associated with the implementation of this set of obesity-prevention policies.

## Background

High intake of added sugars increases the risk of overweight and obesity and several non-communicable diseases [1]. Reducing their consumption is an important target for obesity prevention policies like food labelling or taxation, which can secondarily promote foods and beverages reformulation [2]. One of the main strategies used by the Food Industry to reduce the content of added sugars on packaged foods and beverages, as well as in culinary preparations, is the use of Non-Nutritive Sweeteners (NNS) [3] because they are sweeter than sugar in terms of weight and much less is required to achieve the same level of sweetness, which results in fewer or no calories [4]. NNS are considered food additives and their safety must be evaluated by the Joint FAO/WHO Expert Committee on Food Additives (JECFA) before they can be used [5]. For each approved NNS, there is an acceptable daily intake (ADI) established by JECFA, which is defined as the amount of a substance that can be consumed each day, even over a lifetime, without risk. It is usually expressed as milligrams of the substance per kilogram of body weight per day [6].

There is some concern that children could exceed the ADI for some NNS due to their lower body weight and higher preference for sweet foods and beverages [7]. Moreover, the effect of NNS on health is still controversial. A few cohort studies, mainly based on food frequency questionnaire measurements, have associated NNS consumption with a higher risk of sugar-related non-communicable diseases in adults [8–11], while a prospective study in children has shown a relationship between consumption of artificially-sweetened beverages and weight gain [12]. However, other observational studies have shown no effect and randomized controlled trials in children do not clearly demonstrate NNS’s metabolic effects among the pediatric population [13, 14].

Chile is one of the countries with the highest prevalence of obesity in the world, particularly among children [15]. Most of the population exceeds the World Health Organization recommendation regarding sugars intake and, in adults, 13% of the total energy intake derives from added sugars, mostly from ultra-processed foods [16]. In the past 5 years, Chile has implemented a series of regulatory actions to promote healthier diets, including a sugar-sweetened beverages tax on 2014 [17] and the use of a warning front-of-package labelling in unhealthy products along with marketing restrictions (i.e. Food Labelling and Advertising Law) on June 2016 [18, 19]. These ongoing policies might be promoting foods and beverages reformulation to reduce the content of added sugars by using NNS as replacers. In fact, the Food Industry self-reported the reformulation of 18% of its products on several nutrients (including sugars), between January and June 2016 (before the implementation of the Law) [20], and in a similar period, the largest sugar producer reported a decrease of 20% in its sales, mainly due to a lower purchase by food manufacturers [21]. Considering this new scenario, Chile has not implemented yet any monitoring actions to assess NNS consumption among the Chilean population. This might be an important issue to address given the existing evidence from the United States showing that NNS consumption is increasing among the paediatric and adult population [22].

Consumption of NNS has been positively associated with high socioeconomic status (SES) and educational level [23], while, on the contrary, added sugars consumption has been linked to lower-income groups in the US [24]. Similarly, in Chile, the National Dietary Survey (2010) showed a higher prevalence of artificially-sweetened beverages and table-top sweeteners consumption among high-income adults, while sugar-sweetened beverages and table sugar were more frequently consumed by low-medium SES adults [25]. In this context, we aimed to describe the consumption of six NNS among a cohort of ~ 1000 low-medium income Chilean pre-schoolers from Santiago before the implementation of the Food Labelling Law, identify sociodemographic and anthropometric characteristics associated with their consumption, and describe the main dietary sources of each NNS sub-type. These findings will be relevant for future studies assessing how potential food reformulation prompted by the implementation of the Food Labelling Law might differentially affect NNS consumption among socio-demographic groups.

## Methods

### Subjects and recruitment

This is a descriptive analysis of baseline data from the Food and Environment Chilean Cohort - FEChiC [26–28]. For this cohort study, all public nursery schools located in the South East area of Santiago, Chile (*n* = 55) were invited to participate and 50 accepted to be part of the study. This area covers six low-medium income counties of the city [29], which are similar to low-middle income neighbourhoods from the rest of the country in terms of the level of poverty and nutritional indicators [29, 30].

Recruitment of pre-schoolers and their mothers was carried out between January and April 2016 (before the Food Labelling Law was implemented). All children in pre-kindergarten (aged 4-5y) and kindergarten (aged 5-6y) from the consenting nursery schools were invited to participate (*n* = 2625) and 968 agreed to do so; there were no significant differences between children who agreed to participate in the study compared to the ones that did not, in terms of sex distribution, age, and anthropometric characteristics (*p*-value > 0.05 for all variables). Children with genetic, metabolic or severe chronic diseases that could affect growth or eating behaviour (*n* = 1), pre-schoolers that had a sibling already included in the study (*n* = 3), or those whose mothers declared not being in charge of food purchase or food intake of the child or that were not fluent in Spanish or had a mental illness or a health problem that made it difficult to perform the interviews (*n* = 2) were excluded from the study. Two children left the study before being evaluated. For the purpose of this analysis, participants with incomplete baseline information on food intake, weight, height, and/or sociodemographic background were excluded (*n* = 1). Therefore, the analytical sample size was 959 pre-schoolers aged 4–6-year-old and their mothers.

The sample size calculation for this study (*n* = 959) was performed assuming a 95% confidence level, a variance of 0.06, considering it would allow detecting the average consumption of low-presence NNS (average 0.18 mg/kg/day) with an error of 1.5% [31].

### Ethics

This study was approved by the ethics committee of the Institute of Nutrition and Food Technology, University of Chile. Mothers signed the informed consent on behalf of their children, who were not old enough to give their assent.

### Data collection

From March to June 2016, baseline data collection was carried out at the Institute of Nutrition and Food Technology or at the participants’ house, according to their availability. Food intake data were obtained by trained dietitians using a standardized multi-pass 24-h recall [32] applied to children’s mothers and complemented by the pre-schooler in case of doubts. Briefly, the structured interview enquires about all foods and drinks consumed the previous 24 h using the Five-Step Multiple-Pass Method, which may prompt the participant to remember usually forgotten items [33]. For every item consumed, information about portion size, cooking details, meal occasion (i.e. breakfast, snacking, etc.), meal time, and brand of product (if packaged) was reported and entered into a standardized software. From the 959 24-h recalls, 825 corresponded to weekdays and 134 corresponded to weekends.

Dietary intake data (i.e. type and amount of food items consumed) were then linked to NNS content information from packaged foods. In Chile, there are eight NNS allowed by the Food Sanitary Code including Sodium Cyclamate, Saccharin, Aspartame, Acesulfame Potassium, Sucralose, Steviol glycosides, Alitame and Neotame [34]; however, in the current study, we only considered the first six because Alitame and Neotame were not found among the products consumed by the sample. For each one of them, JECFA established the following ADIs: 0–11 mg/kg body weight/day for Sodium Cyclamate, 0–5 mg/kg body weight/day for Saccharin, 0–40 mg/kg body weight/day for Aspartame, 0–15 mg/kg body weight/day for Acesulfame Potassium and Sucralose, and 0–4 mg/kg body weight/day for Steviol glycosides [35–40]. This information must be declared on the label of packaged foods and beverages that contain them, along with the quantity of NNS in mg per serving size and per 100 g or 100 ml of the product [34].

Since 2015, as part of the INFORMAS-Chile project [41], fieldworkers have collected photos of packaged products from 11 food categories in five supermarkets from high and six from low-middle income neighbourhoods in Santiago, Chile. A standardized protocol described by Kanter et al. was used to guide data collection and management [42]. Briefly, trained dietitians worked in coding nutritional information, including NNS content, of ~ 12,000 packaged foods and beverages that were available on the supermarkets during February 2016 [43]. Based on this information we were able to link NNS content from 99% of the artificially sweetened products reported on the dietary recalls; the remaining 1% (*n* = 17 products) were excluded from the analysis. For some table-top sweeteners (*n* = 88 products) it was not possible to determine whether they contained Steviol glycosides alone or a mixture of Steviol glycosides and other NNS; thus, we had to assume they only contained Steviol glycosides.

For analytical purposes, all artificially sweetened products consumed by the sample were classified into three major dietary sources (i.e. foods, beverages, and table-top sweeteners) and then into eight food groups based on international definitions and culinary practices (see Additional file 1).

Anthropometric measurements were collected on children and their mothers by trained dietitians following standardized procedures (ICC > 0.75 for all measurements, re-checked every 12 months) [44, 45]. Body weight, height, and waist circumference were measured using a digital electronic scale (Seca 770 or 803, precision of 0.1 kg), a portable stadiometer (Seca 217, to the nearest 0.1 cm) and a measuring tape (Lufkin, Model W606PM, with 200 cm capacity and 0.1 cm precision), respectively. All the instruments were calibrated twice a month. Measurements were taken in duplicate and the average value was considered for the analyses. If the difference between the two measurements was above 100 g for weight, 0.7 cm for height, and 0.5 cm for waist circumference [45], a third measure was taken, and the average value was calculated using the two measurements with less discrepancy.

Body Mass Index, defined as weight [kg]/height [m^2^], was calculated for both children and their mothers. Z-scores were used to determine child weight status which was categorized as normal weight (−2SD < BMI-for age z-score < +1SD), overweight (+1SD < BMI-for-age z-score < +2SD) and obese (BMI-for age z-score > +2SD) according to the WHO Child Growth Standards 2006 [46] in children under 5-years-old, and the WHO Growth Reference 2007, in children above 5-years of age [47]. Maternal weight status was classified as non-obese (BMI < 30 kg/m^2^) and obese (BMI > 30 kg/m^2^) based on the WHO cut-off points for Body Mass Index in adults [48].

Waist circumference measurements in children were compared to the USA percentiles described by Fernandez et al. [49] to determine the presence of abdominal obesity using Cook et al.’s criteria (waist circumference above the 90th percentile) [50], while in their mothers, the Adult Treatment Panel III criteria for clinical identification of metabolic syndrome was used (waist circumference above 88 cm) [51].

Sociodemographic data were also collected in the mother’s interview and included child and maternal date of birth, current occupation and level of education, which was categorized as low (less than 12 years of study), medium (12 years of study) and high (more than 12 years of study).

### Data analysis

Categorical variables are presented as percentages and non-normally distributed numerical variables are presented as median and interquartile range (IQR); nonetheless, when presenting NNS intake, we also provided mean and standard deviation (SD) for comparative purposes.

The prevalence of NNS consumption was determined by dietary source, estimating the proportion of pre-schoolers who consumed NNS from: 1) at least one source, 2) food and beverages only, and 3) food, beverages, and table-top sweeteners on the day of the dietary recall. Subsequently, Chi-square independence tests were used to assess the association between NNS consumption and sociodemographic and anthropometric characteristics of the sample, by dietary source. Variables considered for this analysis included some that have previously shown an association with NNS consumption among US paediatric population, such as sex, age, child weight status, and level of maternal education (as a proxy of socioeconomic status) [52], while others, like maternal weight status and the presence of abdominal obesity in children and their mothers, were included for exploratory reasons.

Intakes of the six NNS sub-types were calculated by adding the total amount of each NNS from all food products consumed by each pre-schooler, divided by their weight (kg). Results were presented by sex and Wilcoxon Mann Whitney tests were used to assess differences between females and males.

Finally, dietary sources of NNS were described using the following formula to calculate the contribution of eight food groups (see Additional file 1) to the overall intake of the six NNS sub-types:

$$ {\displaystyle \begin{array}{l}\% of\ contribution\ of< food\ group>\\ {}\  to\kern0.5em the\ overall\ intake\ of< NNS>=\frac{\begin{array}{l}< NNS> intake\ from< food\ group>\\ {} among\  all\  children\end{array}}{\begin{array}{l}< NNS> overall\ intake\ from\  all\  food\ products\\ {}\  among\  all\  children\end{array}}\\ {} among\  all\  children\end{array}} $$

All analyses were carried out with the statistical program STATA 14.0 and a *p*-value < 0.05 was considered statistically significant.

## Results

### Sample characteristics

Table 1 shows the sociodemographic and anthropometric characteristics of the sample. More than half of the pre-schoolers were female, and their median age was 4.7 years. Most of their mothers had a high-school degree or further education (82%) and approximately half of them worked outside their home. Regarding anthropometric characteristics, one out of five pre-schoolers was affected by obesity and 10% presented abdominal obesity, while 1/3 of their mothers were affected by obesity and more than half presented abdominal obesity.

**Table 1:** Sociodemographic and anthropometric characteristics and maternal background of 959 low-medium income pre-schoolers from the Food and Environment Chilean Cohort (FECHIC)

	Total Sample
Female [n (%)]	498 (51.9)
Age, y [median (IQR)]	4.7 (4.4–5.0)
Age of the child’s mother, y [median (IQR)]	31 (26–36)
Level of maternal education [n (%)]	
Low (< 12 years of study)	171 (17.8)
Medium (12 years of study)	397 (41.4)
High (> 12 years of study)	391 (40.8)
Child’s mother works out of home [n (%)]	478 (49.8)
Weight, kg [median (IQR)]	19.2 (17.4–21.6)
Height, cm [median (IQR)]	107.4 (104.2–111.2)
Weight status [n (%)] ^a^	
Normal weight (−2SD < BMI-for age z-score < +1SD)	508 (53.0)
Overweight (+1SD < BMI-for-age z-score < +2SD)	276 (28.8)
Obese (BMI-for-age z-score > +2SD)	175 (18.2)
Waist circumference, cm [median (IQR)] ^b^	54.4 (51.5–57.8)
Children’s abdominal obesity [n (%)] ^bc^	92 (9.6)
Maternal weight status [n (%)] ^de^	
Underweight (BMI <18.5 kg/m^2^)	6 (0.6)
Normal (BMI 18.5–24.9 kg/m^2^)	237 (25.8)
Overweight (BMI 25–29.9 kg/m^2^)	350 (38.1)
Obese (BMI > 30 kg/m^2^)	326 (35.5)
Maternal abdominal obesity [n (%)] ^ef^	509 (55.4)

^a^Defined based on WHO Child Growth Standards 2006 for children under 5-years-old and WHO Growth Reference 2007 for children above 5 years-old [46, 47, 53]

^b^Data were available for 957 preschool children, two children refused to be measured

^c^Waist circumference measures were compared to USA percentiles describe by Fernandez et al. [49] and Cook et al. criteria was used to assess abdominal obesity among children [50]

^d^Defined based on WHO cut-off points for Body Mass Index in adults [48]

^e^Data were available for 919 mothers; 38 mothers were pregnant and two refused to be measured

^f^Defined based on the criteria for clinical identification of metabolic syndrome described by the Adult Treatment Panel III 2001 [51]

### NNS consumption

Results showed that 68% (*n* = 651/959) of the pre-schoolers consumed at least one source of NNS on the day of the dietary recall. Most of them consumed NNS from foods and beverages (*n* = 532/959), while only 12% (*n* = 119/959) also consumed table-top sweeteners (Table 2).

**Table 2:** Prevalence of non-nutritive sweeteners consumption by dietary source among low-medium income pre-schoolers from the Food and Environment Chilean Cohort, by sociodemographic and anthropometric characteristics

	Prevalence of non-nutritive sweeteners consumption, n (%)			
Characteristics	From at least one source of NNS	From foods and beverages only	From foods, beverages and table-top sweeteners	Did not report NNS consumption
**Total sample (*n*** **= 959)**	651 (67.9)	532 (55.5)	119 (12.4)	308 (32.1)
**Sex**				
Female (*n* = 498)	324 (49.8)	259 (48.7)	65 (54.6)	174 (56.5)
Male (*n* = 461)	327 (50.2)	273 (51.3)*	54 (45.4)	134 (43.5)
**Age, years**				
< 4.6 (*n* = 481)	331 (50.8)	275 (51.7)	56 (47.1)	150 (48.7)
> 4.6 (*n* = 478)	320 (49.2)	257 (48.3)	63 (52.9)	158 (51.3)
**Level of maternal education**				
Low (< 12 years of study) (*n* = 171)	107 (16.4)	93 (17.5)	14 (11.8)	64 (20.8)
Medium (12 years of study) (*n* = 397)	258 (39.6)	209 (39.3)	49 (41.2)	139 (45.1)
High (12 years of study) (*n* = 391)	286 (44.0)*	230 (43.2)*	56 (47.0)*	105 (34.1)
**Child weight status**^a^				
Normal weight (*n* = 508) (−2SD < BMI-for age z-score < +1SD)	335 (51.5)	267 (50.2)	68 (57.1)	173 (56.2)
Overweight (*n* = 276) (+1SD < BMI-for-age z-score < +2SD)	191 (29.3)	172 (32.3)	19 (16.0)	85 (27.6)
Obese (*n* = 175) (+1SD < BMI-for-age z-score < +2SD)	125 (19.2)	93 (17.5)	32 (26.9)**	50 (16.2)
**Child abdominal obesity**^**bc**^				
Absence (*n* = 865)	582 (89.5)	482 (90.8)	100 (84.0)	283 (92.2)
Presence (*n* = 92)	68 (10.5)	49 (9.2)	19 (16.0)*	24 (7.8)
**Maternal weight status**^**de**^				
Non obese (BMI < 30 kg/m^2^) (*n* = 593)	412 (66.2)	333 (65.7)	79 (68.7)	181 (60.9)
Obese (BMI > 30 kg/m^2^) (*n* = 326)	210 (33.8)	174 (34.3)	36 (31.3)	116 (39.1)
**Maternal abdominal obesity**^**ef**^				
Absence (*n* = 410)	287 (46.1)	228 (45.0)	59 (51.3)	123 (41.4)
Presence (*n* = 509)	335 (53.9)	279 (55.0)	56 (48.7)	174 (58.6)

^a^Defined based on WHO Child Growth Standards 2006 for children under 5-years-old and WHO Growth Reference 2007 for children above 5-years-old [46, 47, 53]

^b^Waist circumference measures were compared to USA percentiles describe by Fernandez et al. [49] and Cook et al. criteria was used to assess abdominal obesity among children [50]

^c^Data were available for 957 preschool children, two children refused to be measured

^d^Defined based on WHO cut-off points for Body Mass Index in adults [48]

^e^Data were available for 919 mothers; 38 mothers were pregnant and two refused to be measured

^f^Defined based on the criteria for clinical identification of metabolic syndrome described by the Adult Treatment Panel III 2001 [51]

* *P* < 0.05 analysed using Chi^2^ independence test

** *P* < 0.01 analysed using Chi^2^ independence test

The prevalence of NNS consumption was significantly associated with the level of maternal education (*p* < 0.05). A higher proportion of children whose mothers had a high level of education consumed NNS compared to the ones whose mothers had lower levels of education. This result was consistent for all children reporting NNS consumption on the day of the dietary recall regardless of the source.

Whilst the prevalence of NNS consumption from at least one source did not differ by sex or other anthropometric or maternal characteristics (*p* > 0.05), our results showed that a greater proportion of boys consumed NNS from foods and beverages compared to girls (*p* < 0.05), and that the prevalence of obesity and abdominal obesity was higher among children who reported NNS consumption, including table-top sweeteners, compared to the ones that did not report NNS consumption (*p* < 0.05) (Table 2).

Regarding NNS’s intake, Aspartame showed the highest intake [2.5 (1.4–3.7) mg/kg of body weight per consumer], followed by Sodium Cyclamate [1.6 (1.3–2.6) mg/kg of body weight per consumer] and Steviol glycosides [1.2 (0.2–2.1) mg/kg of body weight per consumer]. Non-significant differences were observed by sex (*p* > 0.05) (Table 3).

**Table 3:** Non-nutritive sweeteners intakes among low-medium income pre-schoolers from the Food and Environment Chilean Cohort, by sex

				Non-nutritive sweeteners consumers						
	Total			Girls			Boys			***P***-value
	n	Median (IQR)	Mean (SD)	n	Median (IQR)	Mean (SD)	n	Median (IQR)	Mean (SD)	
**Sodium Cyclamate**	9	1.6 (1.3–2.6)	2.0 (1.1)	5	1.3 (0.8–1.3)	1.6 (1.3)	4	2.4 (1.9–3.0)	2.4 (0.8)	0.142
**Saccharin**	12	0.7 (0.3–1.2)	0.8 (0.5)	6	0.3 (0.2–0.8)	0.5 (0.4)	6	1.0 (0.6–1.3)	1.0 (0.4)	0.078
**Aspartame**	241	2.5 (1.4–3.7)	3.1 (2.6)	115	2.4 (1.4–3.4)	2.8 (2.0)	126	2.8 (1.4–4.2)	3.4 (3.0)	0.260
**Acesulfame Potassium**	268	0.9 (0.6–1.8)	1.4 (1.4)	126	0.9 (0.5–1.7)	1.2 (1.1)	142	1.0 (0.6–1.9)	1.5 (1.6)	0.277
**Sucralose**	520	0.9 (0.4–1.5)	1.1 (1.0)	248	0.9 (0.4–1.6)	1.1 (1.0)	272	0.8 (0.5–1.5)	1.1 (1.0)	0.747
**Steviol glycosides**	216	1.2 (0.2–2.1)	1.5 (1.9)	109	1.2 (0.2–2.1)	1.6 (2.3)	107	1.2 (0.3–2.0)	1.5 (1.5)	0.910

Non-nutritive sweeteners intakes are expressed in mg/kg of body weight per consumer.

Wilcoxon-Mann-Whitney tests were used to compare NNS consumption by sex. *P*-value < 0.05 was considered statistically significant.

The consumption of almost all NNS did not show a statistically significant variation according to the day of the dietary recall. The only exception was seen for Aspartame, whose median intake on weekdays reached 2.6 (1.5–3.7) mg/kg of body weight per consumer, while on weekends it was 1.7 (1.1–3.1) mg/kg of body weight per consumer (*p*-value < 0.05) (data not shown).

Further analysis showed that 13 pre-schoolers (1% of the sample) potentially exceeded the ADI for Steviol glycosides on the day of the dietary recall (data not shown).

### Dietary sources of NNS

**Fig. 1:**
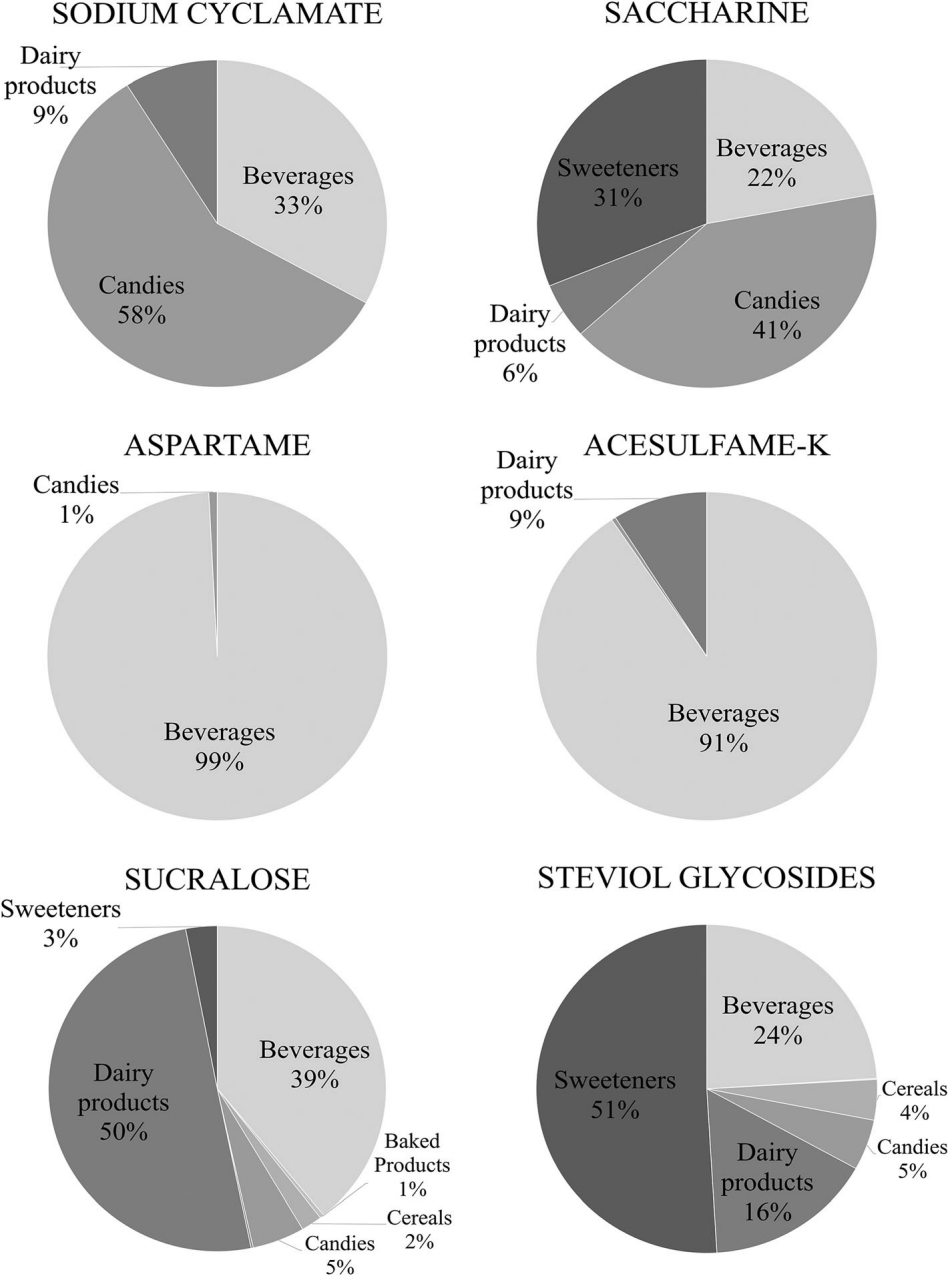
Contribution of dietary sources to the intake of six Non-Nutritive Sweeteners among Chilean pre-schoolers (*n* = 651consumers). Total intakes of each NNS were: Sodium Cyclamate = 326.5 mg/day; Saccharine = 164.7 mg/day; Aspartame = 14,397.4 mg/day; Acesulfame-K = 7237.9 mg/day; Sucralose = 11,443.4 mg/day & Steviol glycosides = 6658.7 mg/day)

From the eight food groups analysed (see Additional file 1), beverages were the top contributors to the intake of Aspartame (99%) and Acesulfame Potassium (91%); moreover, beverages were the only food group that contributed to the intake of all six NNS studied. Candies (specifically the subgroup jellies [results not shown]) were the top contributors to Sodium Cyclamate and Saccharine intake (58 and 41%, respectively), while dairy products were the main dietary source of Sucralose, accounting for 50% of its total intake. Finally, half of Steviol glycosides intake was due to table-top sweeteners consumption (Fig. 1).

## Discussion

In a sample of low-medium income pre-schoolers from an ongoing longitudinal study taking place in Santiago, Chile we found that the majority of children (68%) consumed NNS before the implementation of the Food Labelling and Advertising Law [19]. This result is in line with studies conducted in older Chilean children (6 to < 18y) reporting that 75 to 100% of the sample consume NNS [54, 55]. However, international evidence has shown a lower prevalence of NNS consumption. Sylvetsky et al. [52] reported that in 2009–2010 and 2011–2012, 22% of US pre-schoolers (2-5y) consumed NNS in at least one of the two 24-h recall analysed while Grech et al. [56] described that in 2011–12 less than 5% of Australian children (4–8-year-old) consumed NNS (based on a single 24-h recall).

Our results showed that 12% of the pre-schoolers consumed table-top sweeteners on the day of the dietary recall. This prevalence is considerably higher than the 8% reported among 2–5-year-old children in the Chilean National Dietary Survey conducted in 2010–2011 based on a food-frequency questionnaire [25]. Several factors such as sample characteristics and measurement methods used to collect dietary intake can explain these differences; however, these results may also indicate that consumption of table-top sweeteners is possibly increasing among Chilean children and, therefore, monitoring actions should be emphasized at a national level using consistent methods to avoid study design-related differences.

Only a few studies have assessed sociodemographic and anthropometric determinants of NNS consumption [52, 56]. Sylvetsky et al. described that a significantly greater proportion of high SES US children (2 to < 18y) consumed NNS than those with lower SES [52]. Similarly, in our study, the prevalence of NNS consumption was significantly higher among children whose mothers had a high level of education compared to the ones whose mothers had a lower level of education. This variable can be considered a good proxy of SES as it was defined by mothers’ grade level, which is an important moderator in the correlation between SES and academic achievement [57]. A recent study published by our group reported that Chilean mothers from all SES are changing their food attitudes and behaviours as a result of the implementation of the Food Labelling Law [58], highlighting the need of monitoring the impact of these changes on food behaviours as well. Sylvetsky et al. also reported a significantly higher prevalence of NNS consumption among American children (2 to <18y) who were affected by obesity compared to the ones with overweight or normal weight status [52]. In our study we did not find significant differences in the overall prevalence of NNS consumption by weight status; however, we showed that in users of table-top sweeteners, the prevalence of obesity and abdominal obesity was much higher than in non-NNS consumers (*p* < 0.05). Previous studies in Chilean school-aged children (6 to <18y) have reported a higher consumption of Acesulfame K, Aspartame and Sucralose in children affected by obesity compared to those with normal weight status [54, 55]. Interestingly, we and other studies conducted in paediatric population have not detected significant differences in the overall prevalence of NNS consumption by sex [52, 56].

There is little evidence of the type and quantity of NNS that children at this age consume [31]. Martyn et al. assessed the consumption of Aspartame, Acesulfame K, Saccharine, and Sucralose in a representative sample of Irish pre-schoolers (1-4y) using a 4-day weighted food diary [31]. In line with our results, Aspartame was the most consumed NNS; however, Martin et al. reported lower intakes of the four NNS studied compared to our research [31]. Furthermore, NNS daily intakes among Irish pre-schoolers did not exceed the ADIs, even among high-level consumers (P95) [31], whereas our study found that 2% of the pre-schoolers consuming NNS (*n* = 13/651) potentially exceeded the ADI for Steviol glycosides on the day of the dietary recall. This estimation could have been overestimated due to the fact that for some table-top sweeteners (*n* = 88) we had to assume that they only contained Steviol glycosides, as there was not enough information to know if they contained a mixture of Steviol glycosides and other NNS. Although a single exposure above the ADI may not represent a health concern, this is a worrying scenario for Chile, as it could be expected that the percentage of pre-schoolers exceeding the ADI for Steviol glycosides increases after the implementation of the Food Labelling and Advertising Law [19]. This set of policies could stimulate food and beverage reformulation including the replacement of added sugars by NNS [2]. On the other hand, the use of NNS mixtures could also increase in order to lower individual exposure to each NNS sub-type [59], which could be potentially beneficial; subsequent follow-ups of this cohort and other studies will allow testing some of these hypotheses.

Regarding dietary sources, our research showed that beverages were the only food group that contributed to the consumption of the six NNS studied, being the most important source of Aspartame and Acesulfame potassium among our sample of pre-schoolers. These findings are in line with results reported by Martyn et al. in Irish pre-schoolers [31].

The present study is not exempt from limitations. One key limitation is that our sample only comprised low-medium income pre-schoolers from Santiago, the capital of the country. In Chile, almost 90% of the population is urban [60] and the participants of our study were from neighbourhoods that have similar levels of poverty and nutritional indicators than low-middle income neighbourhoods from the rest of the country [29, 30]; moreover, our estimates are based on a convenience sample given that the studied pre-schoolers are participants of an ongoing longitudinal study. Another limitation is the use of a single 24-h recall to assess NNS consumption, which might not be representative of usual intakes due to day-to-day variation [61, 62]; nonetheless, some studies have described that a single survey could be used to estimate average intakes [61, 63]. Recall bias is another limitation that has to be considered. We have explored misreporting of dietary intake in a similar cohort and results have shown that it is mostly driven by a low report of foods high in sugars and saturated fats [64]; therefore, we do not believe misreporting could significantly affect our results.

As described in our methodology, most of our dietary information was from weekdays (*n* = 825/959). However, the only significant difference in NNS consumption by the day of the dietary recall was found for Aspartame, whose median intake on weekdays was higher than on weekends (*p*-value < 0.05). On the other hand, the assumption that some table-top sweeteners contained Steviol glycosides alone instead of a mixture of Steviol glycosides and other NNS (*n* = 88) could have led to a slight overestimation of Steviol glycosides intake; however, only three of the 13 pre-schoolers who exceed the ADI for Steviol Glycosides consumed table-top sweeteners as their only source of NNS while five children did not consume table-top sweeteners at all. We did not find NNS concentration data only on a few table-top sweeteners containing Saccharine or Aspartame (*n* = 17). Thus, we believe that the impact of these omissions was low since they accounted for less than 2% of artificially sweetened products consumed by the sample.

Among the strengths of this research, it can be mentioned that the use of a standardized multi-pass 24-h recall provided detailed information about food products consumed by the sample (including names and brands), which allowed us to link this information to the corresponding NNS content data in 99% of the products. This enabled us to characterize NNS’s intake among our sample with appropriate validity. Additionally, information collected regarding sociodemographic and anthropometric characteristics of the children and their mothers allowed us to identify potential groups of children with a higher prevalence of NNS consumption.

## Conclusion

Before the implementation of the Food Labelling and Advertising Law in Chile, NNS consumption was highly prevalent among pre-schoolers from the Food and Environment Chilean Cohort, especially among the ones whose mothers had a high level of education. Since the law was implemented, several reports suggest that food products and beverages have been reformulated. Future follow-up of FECHIC participants will allow us to assess whether the Food Labelling Law has impacted NNS consumption among this sample of Chilean children. Altogether, we believe these results highlight the importance of monitoring NNS’s intake among the paediatric population, especially in those with higher educational level and with higher beverage intake.

## Supplementary information

**Additional file 1.** Food groups defined for packaged products consumed by a sample of Chilean pre-schoolers for which non-nutritive sweeteners were identified.

## Data Availability

The datasets generated and/or analysed during the current study are not publicly available because the main cohort study is still ongoing but are available from the corresponding author on reasonable request.

## Abbreviations

NNS: Non-Nutritive Sweeteners
ADI: Acceptable daily intake
JECFA: Joint FAO/WHO Expert Committee on Food Additives
SES: Socioeconomic status
