# Correction to: Consumption of non-nutritive sweeteners by pre-schoolers of the food and environment Chilean cohort (FECHIC) before the implementation of the Chilean food labelling and advertising law

**DOI:** 10.1186/s12937-020-00655-4

**Published:** 2020-12-05

**Authors:** Carolina Venegas Hargous, Marcela Reyes, Lindsey Smith Taillie, Carmen Gloria González, Camila Corvalán

**Affiliations:** 1 Institute of Nutrition and Food Technology, University of Chile, Av. El Líbano 5524, Macul, Casilla, 138-11 Santiago, Chile; 2 Carolina Population Center, University of North Carolina at Chapel Hill, 123 West Franklin St., Suite 210, Chapel Hill, 27516 NC United States

**Correction to: Nutr J 19, 69 (2020)**

**https://doi.org/10.1186/s12937-020-00583-3**

Following publication of the original article [1], the authors realized that one of the funding sources for this study was left out of the funding section.

The updated Funding section is given below and the changes have been highlighted in **bold typeface**.

This work was supported by Regular FONDECYT, CONICYT [grant number 161436] and by the International Development Research Centre -IDRC- [grant number 108180–001 & 107731–002]. **FONIS SA19I0128 (PI M. Reyes) financed the open-access for this paper.** Funders did not have any influence in the study design, data collection, analysis or interpretation of the data, nor in writing the manuscript or the decision to submit the paper for publication.

The original article [1] has been corrected.
