# Supplementary material for: Consumption of non-nutritive sweeteners by pre-schoolers of the food and environment Chilean cohort (FECHIC) before the implementation of the Chilean food labelling and advertising law
Source: Nutr J. 2020 Jul 10;19:69. doi: 10.1186/s12937-020-00583-3 (PMC7353755; doi:10.1186/s12937-020-00583-3)
Supplement: Supplementary file 1 — Additional file 1. Food groups defined for packaged products consumed by a sample of Chilean pre-schoolers for which non-nutritive sweeteners were identified. [file 12937_2020_583_MOESM1_ESM.docx]

**Additional file 1.** Food groups defined for packaged products consumed by a sample of Chilean pre-schoolers for which non-nutritive sweeteners were identified.

| **Food Group** | **Food Sub-group** | **Types of foods included** |
| --- | --- | --- |
| Beverages | Nectars | Nectars |
|  | Flavoured waters | Flavoured carbonated and non-carbonated waters |
|  | Other beverages | Powder juice concentrates and Juice drinks (up to 24% juice) |
|  | Soft drinks | Cola and non-cola carbonates |
|  | Vegetable drinks | Soy Beverages |
| Baked products | Bread | Packaged bread |
|  | Sweet biscuits | Sweet biscuits without filling and whole grain cookies |
| Cereals | Breakfast cereals | Children breakfast cereals (flakes, balls, hearts, etc.) |
| Candies | Jellies | Jellies and gummy bears |
|  | Bubble gums | Bubble gums |
| Sweet spreads | Jams | Fruit jams |
| Dairy products | Yogurts | Yogurts, liquid yogurt, fermented milk drinks, yogurts with fruits or cereals |
|  | Milk drinks | Milk drinks with or without probiotics |
|  | Milks | Flavoured skimmed or semi-skimmed milk |
|  | Milk-based desserts | Flan, rice milk pudding, semolina milk pudding |
| Processed fruits | Fruit compote | Fruit compote |
| Sweeteners | Non-caloric sweeteners | Table-top non-caloric sweeteners |
|  | Caloric sweeteners | Light sugar, milk flavourings |
